# Pemetrexed induces ROS generation and cellular senescence by attenuating TS‐mediated thymidylate metabolism to reverse gefitinib resistance in NSCLC

**DOI:** 10.1111/jcmm.17799

**Published:** 2023-06-06

**Authors:** Yun Chen, Chen Zhang, Shidai Jin, Jun Li, Jiali Dai, Zhihong Zhang, Renhua Guo

**Affiliations:** ^1^ Department of Oncology Jiangsu Province People's Hospital and Nanjing Medical University First Affiliated Hospital Nanjing China; ^2^ Department of Pathology Jiangsu Province People's Hospital and Nanjing Medical University First Affiliated Hospital Nanjing China

## Abstract

Epidermal growth factor receptor tyrosine kinase inhibitors (EGFR‐TKI) are strongly recommended for non‐small‐cell lung cancer (NSCLC) patients harbouring active EGFR mutations, while drug resistance makes exploring resistance mechanisms and seeking effective therapeutic strategies urgent endeavours. Thymidylate synthetase (TYMS or TS) is a dominant enzyme in thymidylate nucleotide metabolism. In this study, we found a positive correlation between TS expression and overall survival (OS) and disease‐free survival (DFS) in lung adenocarcinoma. The examination of gene sets from 140 NSCLC patients received EGFR‐TKI therapy demonstrated a negative correlation between high TS expression and the efficacy of EGFR‐TKI therapy. 24 tissue specimens from NSCLC patients exhibited upregulated TS mRNA expression in NSCLC patients resistant to gefitinib. The NSCLC cell PC9 and HCC827 sensitive to gefitinib and relatively resistant PC9/GR and HCC827/GR cells were used to demonstrate the knockdown of TS restored the sensitivity of resistant cells to gefitinib. Furthermore, pemetrexed effectively suppressed TS‐mediated thymidylate metabolism and induced ROS generation, DNA damage and cellular senescence, thereby hampering cancer progression and restoring sensitivity to gefitinib. Our findings illuminate the potential mechanism of TS‐triggered gefitinib resistance and indicate inhibition of TS by pemetrexed can potentiate the effect of gefitinib in NSCLC. Pemetrexed combined with gefitinib has potent anti‐progression potential in gefitinib‐resistant NSCLC. This study suggests that NSCLC patients with both high TS expression and EGFR‐driving mutations might benefit more from a combination strategy of EGFR‐TKI and pemetrexed‐based chemotherapy than EGFR‐TKI monotherapy, which has profound clinical implications and therapeutic value.

## 1 INTRODUCTION

As reported, there were almost 2.2 million new lung cancer cases and 1.8 million related deaths in 2020, and lung cancer remains the predominant cause of cancer‐related deaths worldwide.^1^ Non‐small‐cell lung cancer (NSCLC) accounts for the vast majority of all pathological classifications in malignant lung carcinomas.^2^, ^3^ Epidermal growth factor receptor (EGFR) is a transmembrane receptor tyrosine kinase that plays a significant role in the development and progression of NSCLC.^4^ NSCLC patients with attractive EGFR driver gene mutations are strongly recommended to receive EGFR tyrosine kinase inhibitors (EGFR‐TKI), such as gefitinib, erlotinib, and afatinib. EGFR‐TKI result in considerable improvements in disease control and overall survival in advanced NSCLC. Additionally, a significantly improved quality of life is credited to minor adverse effects of EGFR‐TKI compared to traditional chemotherapy.^5^, ^6^, ^7^, ^8^, ^9^ Undeniably, despite the excellent curative effect of EGFR‐TKI, a fair proportion of NSCLC patients inevitably suffer from drug resistance within 9–13 months.^10^, ^11^ The most common mechanism of resistance is a threonine‐to‐methionine amino acid change at position 790 (T790M) of exon 20. This mutation accounts for approximately 50%–60% of cases with acquired resistance to first‐line EGFR‐TKI, and osimertinib is a specific proven agent aimed at this gatekeeper mutation.^12^, ^13^ Other mechanisms include second point mutations, such as D761Y,^14^ T854A^15^ or L747S,^16^ and histological and phenotypic transformations, such as SCLC transformation and epithelial–mesenchymal transition (EMT). However, several underlying mechanisms of resistance to EGFR‐TKI remain complicated and largely elusive.^17^, ^18^, ^19^ Exploring novel therapeutic strategies for reversing drug resistance in lung cancer has been a long‐standing topic.

Of note, stress from the tumour microenvironment triggers alterations in metabolic preferences in cancer cells to fulfil the increased nutrient consumption required for cell survival and proliferation, which is so‐called ‘metabolic reprogramming’.^20^, ^21^ Otto Warburg first observed that cancer cells utilize aerobic glycolysis as their preferable energy resource rather than mitochondrial oxidative phosphorylation, which provides the vital energy supply in normal cells. This phenomenon in cancer cells is well known as the ‘Warburg effect’.^22^ Since then, strategies targeting tumour metabolic reprogramming have attracted considerable attention from many researchers. Targeting hexosamine biosynthesis induces metabolic vulnerabilities in KRAS/LKB1 co‐mutant lung cancer and subsequently inhibits cancer proliferation.^23^ Impairment of mitochondrial metabolism induced by targeting BACH1 can make triple‐negative breast cancer (TNBC) more sensitive to chemotherapy and targeted therapy.^24^ Enhancement of antioxidant capacity and nucleotide precursors triggers oncogenic transformation.^25^

Pemetrexed, a TS‐targeted agent, exerts anticancer efficacy by inducing thymidylate deficiency and imbalances in the intracellular nucleotide pool.^26^, ^27^ Our previous study demonstrated that EGFR‐TKI combined with pemetrexed considerably improved PFS in advanced NSCLC patients carrying EGFR driving mutations.^28^ However, how to determine the specific population benefit from such combination therapy under the comprehensive assessment of adverse effects and clinical efficacy? It is urgently needed to be explored to seek out new biomarkers and therapeutic targets to help guide clinical decisions. In this study, we first describe the baseline characteristics and the expression level of TS in NSCLC with attractive EGFR mutations.^29^ Furthermore, we demonstrated that TS‐mediated thymidylate metabolism drives tumorigenesis and generates gefitinib resistance in NSCLC. Additionally, the potential mechanism by which pemetrexed induces metabolic vulnerability and reverses gefitinib resistance was explored and elucidated.

## 2 RESULTS

### 2.1 The level of TS in LUAD and multiple other solid malignancies is significantly upregulated and correlated with decreased disease‐free survival (DFS) and overall survival (OS)

**FIGURE 1:**
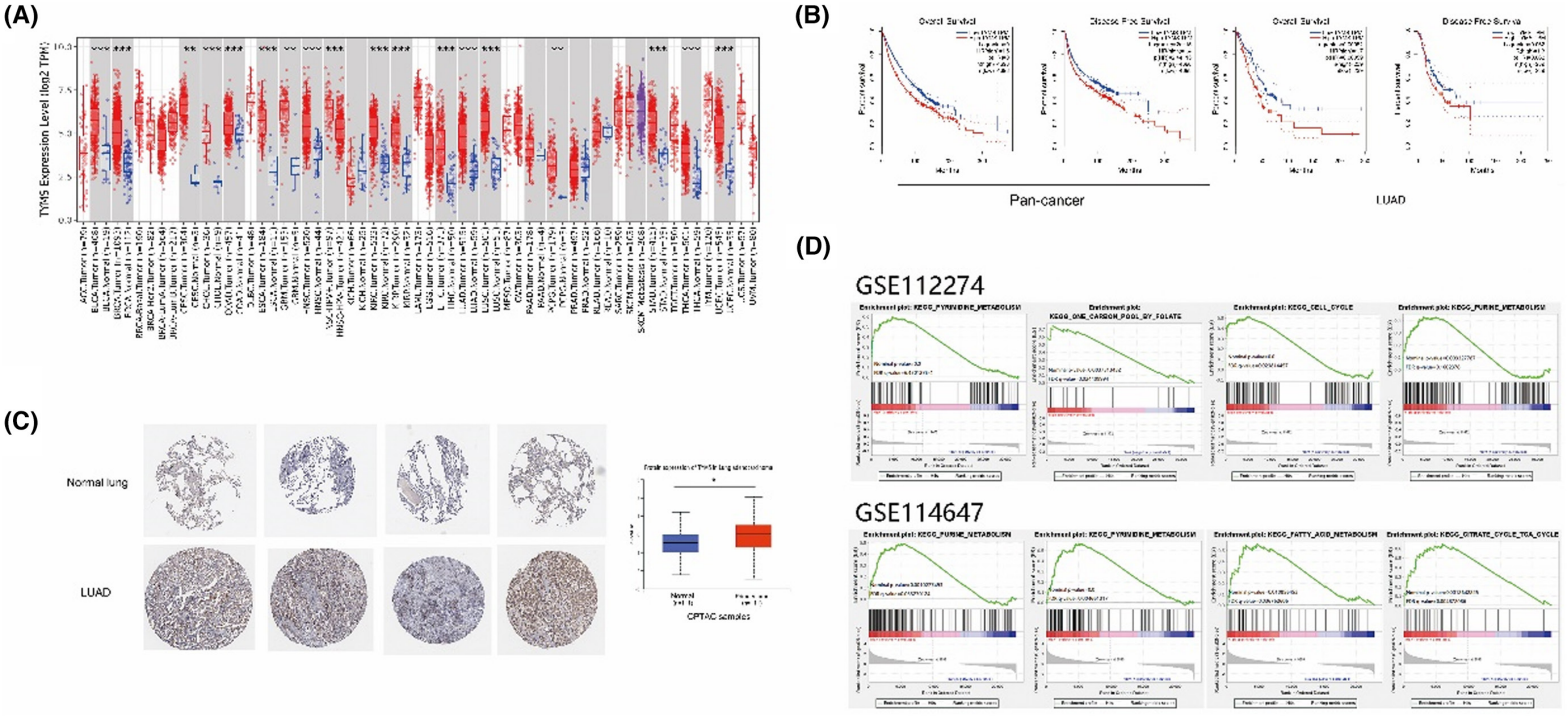
The level of TS in LUAD and multiple other solid malignancies is significantly upregulated and correlated with decreased disease‐free survival (DFS) and overall survival (OS). (A), Thymidylate synthetase (TYMS or TS) mRNA expression in multiple cancer types. (B), Kaplan–Meier survival analysis comparing high and low TS expression in both the pan‐cancer cohort and the lung adenocarcinoma (LUAD) cohort in GEPIA. (C), The protein level of TS in LUAD tissues and adjacent normal lungs. (D), The metabolic pathways upregulated in gefitinib‐resistant datasets (GSE112274 and GSE114647) with high TS expression, displayed with gene set enrichment analysis (GSEA) using GSEA software. *p < 0.05.

First, we performed a pan‐cancer analysis in over 4000 primary tumour samples of 31 common malignant carcinomas in the ONCOMINE database (www.oncomine.org). We found that TS mRNA expression was considerably upregulated in tumour samples compared with nontumor controls, except for acute myeloid leukaemia (LAML) (Figure 1A). Based on this result, with Gene Expression Profiling Interactive Analysis (GEPIA),^30^ we further analysed the importance of the TS gene in DFS and OS in the above‐related 30 tumour types, of which LAML was excluded due to its lower TS expression in contrast with normal controls. As expected, cancer patients with higher TS expression levels had shorter DFS (Figure 1B, HR 1.4, log‐rank *p* < 0.001) and OS (Figure 1B, HR 1.5, log‐rank *p* < 0.001). To explore the significance of TS in lung cancer, we further demonstrated an upregulation trend of TS protein expression in lung adenocarcinoma samples compared to normal samples in the Human Protein Atlas (HPA) database (Figure 1C). Furthermore, high TS expression was correlated with unfavourable DFS (Figure 1B, HR 1.3, log‐rank *p* = 0.062) and OS (Figure 1B, HR 1.7, log‐rank *p* < 0.001) in LUAD. Based on the above analyses, we focused on the function of TS in EGFR‐TKI resistance. The gene expression profile of gefitinib‐resistant samples in two datasets (GSE114647 and GSE112274) was extracted for further analysis. The resistant samples were retrospectively redivided into two groups (TS‐high group and TS‐low group) according to the median expression level and subsequently subjected to multiple gene set enrichment analysis (GSEA) using GSEA software. As suspected, the pyrimidine metabolic pathway and purine biosynthesis pathway were upregulated in the two TS‐high groups, and several other pathways, such as fatty acid metabolism, the TCA cycle and one carbon pool, were also activated in the TS‐high group (Figure 1D). All the above results indicate that TS might serve as a novel molecular marker for the prediction of disease progression and patient outcome in LUAD and many malignant tumours and may act as an effective marker to predict EGFR‐TKI resistance in LUAD.

### 2.2 Patients with upregulated TS expression have lower PFS during EGFR‐TKI therapy

**FIGURE 2:**
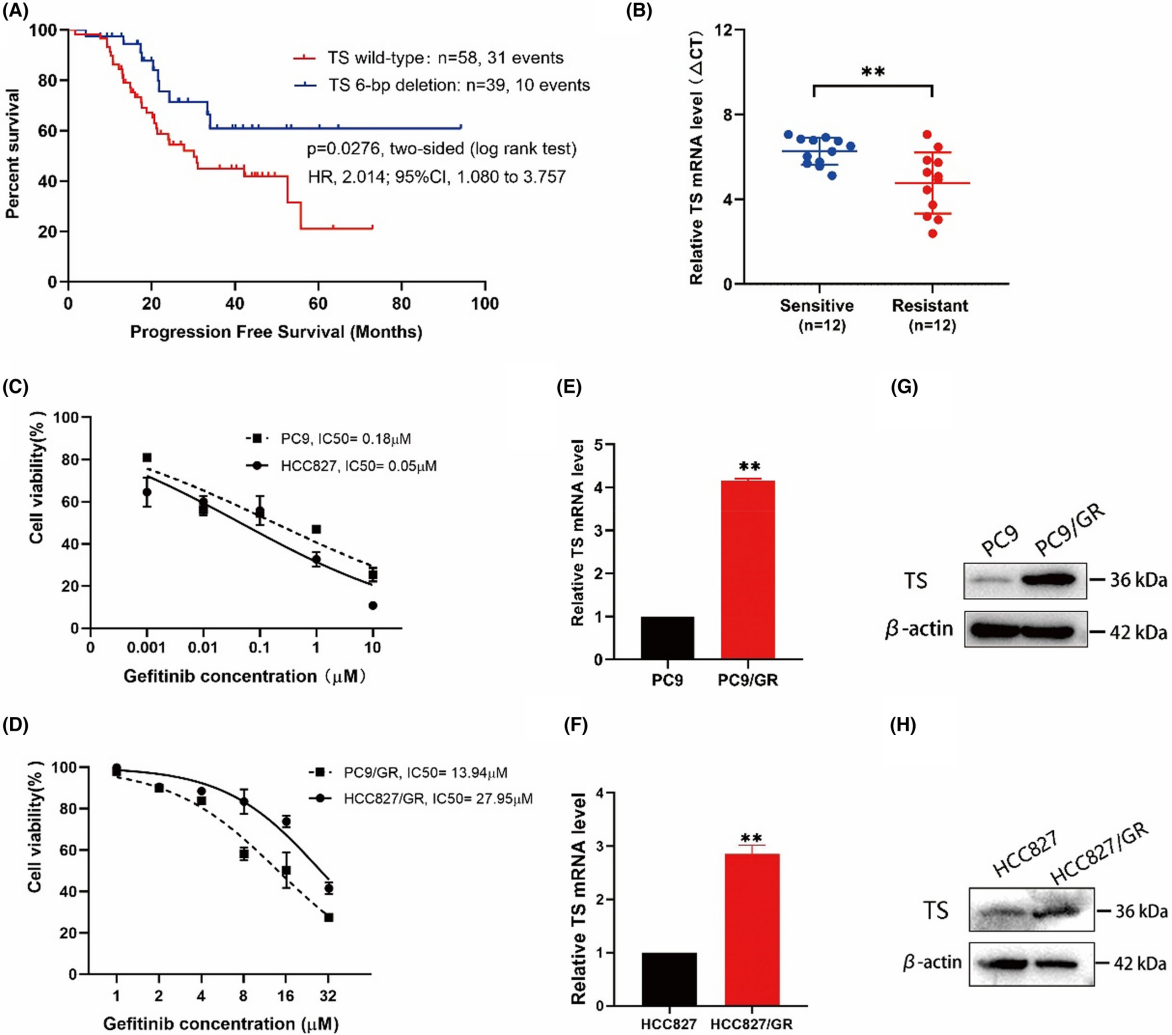
High expression of TS is correlated with efficacy of EGFR‐TKI therapy. (A), Kaplan–Meier survival analysis of PFS in 97 LUAD patients accepting EGFR‐TKI treatment, comparison of the TS wild‐type group and the 6‐bp deletion group. (B), TS expression in NSCLC tissues from NSCLC patients who had never been administered gefitinib (sensitive group) compared to patients who developed resistance during gefitinib treatment (resistant group). The TS expression level in two groups was measured using qPCR and normalized to GAPDH expression. The ΔCt value was calculated by subtracting the GAPDH Ct value from the TS Ct value. Smaller ΔCt values represent higher TS mRNA levels. (C), the IC50 value of gefitinib in PC9 and HCC827 cells. (D), the IC50 value of gefitinib in PC9/GR and HCC827/GR cells. (E, F), qRT–PCR showing the mRNA expression of TS in sensitive and resistant cells. (G, H), the protein level of TS in sensitive and resistant cells. The results represented the average of three independent experiments. **p < 0.01.

First, we retrospectively analysed the correlation between TS polymorphism variation and the efficacy of EGFR‐TKI therapy in NSCLC patients. Between November 2014 and December 2021, a total of 140 patients pathologically confirmed to have NSCLC harbouring EGFR driving mutations were included in the study, among which 68 (49%) patients had wild‐type TS, and the remaining 72 (51%) patients were confirmed to have different TS polymorphism variations. Given previous studies have demonstrated that the 3R/3R genotype is usually accompanied by a relatively higher TS mRNA level than the TS wild type, while the 6‐bp deletion in the 3'UTR of the TS gene promoter region is correlated with a lower TS mRNA level,^31^, ^32^ patients with co‐mutation of 3R/3R and 6‐bp deletion were excluded from our analysis because the uncertainty of TS mRNA level induced by 3R/3R and 6‐bp deletion co‐mutations. Patients with only the 3R/3R genotype were also not included here because the sample size was too small to be representative (only 6 patients harboured the 3R/3R genotype). To analyse the relationship between the TS mRNA level and EGFR‐TKI‐targeted therapy, 10 patients with wild‐type TS and 2 patients with 6‐bp deletion were excluded due to a lack of EGFR‐TKI treatment information. Finally, 58 patients with wild‐type TS and 39 patients with a 6‐bp deletion were followed for Kaplan–Meier analysis of progression‐free survival (PFS). Figure 2A showed that 31 (53.45%) patients were observed to have disease progression in the TS wild‐type group, whereas 10 (25.64%) events occurred in the TS 6‐bp deletion group. The median PFS in the TS wild‐type group was 21.25 months versus 35.77 months in the TS 6‐bp group (HR 2.014, log‐rank *p* = 0.02746, 95% CI, 1.080–3.757). The result suggested that the upregulated expression of TS is correlated with a worse efficacy of EGFR‐TKI therapy. Patient details and clinical characteristics are listed in Table 1.

**TABLE 1:** Patient clinicopathological characteristics and TS polymorphism variant analysis in 140 NSCLC patients harbouring driver EGFR mutations.

Characteristics		TS polymorphism, *n* (%)			
		Wild type	3R	3R + 6bp‐	6bp‐
		68 (49)	6 (4)	25 (18)	41 (29)
Sex, *n* (%)	Male	27 (19)	4 (3)	5 (4)	14 (10)
	Female	41 (29)	2 (1)	20 (14)	27 (19)
Age, years, *n* (%)	<65	46 (33)	5 (4)	18 (13)	22 (16)
	≥65	21 (15)	1 (1)	7 (5)	20 (14)
Stage, *n* (%)	I	18 (13)	3 (2)	6 (4)	9 (6)
	II	5 (4)	0 (0)	1 (1)	3 (2)
	III	3 (2)	11 (8)	2 (1)	5(4)
	IV	39 (28)	2 (1)	15 (11)	19 (14)
	NA	2 (1)	0 (0)	1 (1)	6 (4)
EGFR mutation, *n* (%)	19DEL	25 (18)	2 (1)	11 (8)	16 (11)
	21L858R	33 (24)	3 (2)	11 (8)	22 (16)
	19DEL and 21L858R	0 (0)	0 (0)	0 (0)	1 (1)
	Rare mutations	8 (6)	1 (1)	3 (2)	4 (3)

### 2.3 High TS expression is associated with gefitinib resistance in NSCLC

Based on the above observation, we determined the expression of TS in biopsy specimens from 24 patients with pathologically confirmed NSCLC harbouring EGFR‐sensitive mutations, such as EGFR exon 19 deletion (19DEL) or exon 21 mutation (L858R), was examined. Patients who had never received gefitinib were included in the gefitinib‐sensitive group, and patients experiencing disease progression during or after treatment with gefitinib were placed in the gefitinib‐resistant group. qRT‐PCR assays showed that patients in the gefitinib‐resistant group had a tremendous increase in TS expression compared to patients in the gefitinib‐sensitive group (Figure 2B). After that, we further determined the TS expression level in gefitinib‐sensitive lung cancer cells (PC9 and HCC827) and the corresponding gefitinib‐resistant PC9/GR and HCC827/GR cells. Before that, we first detected the IC50 values of gefitinib in this four cell lines (Figure 2C,D). Next, qRT‐PCR (Figure 2E,F) and WB (Figure 2G,H) experiments were used to demonstrat that both mRNA and protein levels of TS were higher in resistant cells than that in sensitive cells. Thus, the results of both NSCLC patients resistant to EGFR‐TKI and cell lines suggested that the upregulation of TS induces gefitinib resistance.

### 2.4 TS overexpression promotes gefitinib resistance in NSCLC

**FIGURE 3:**
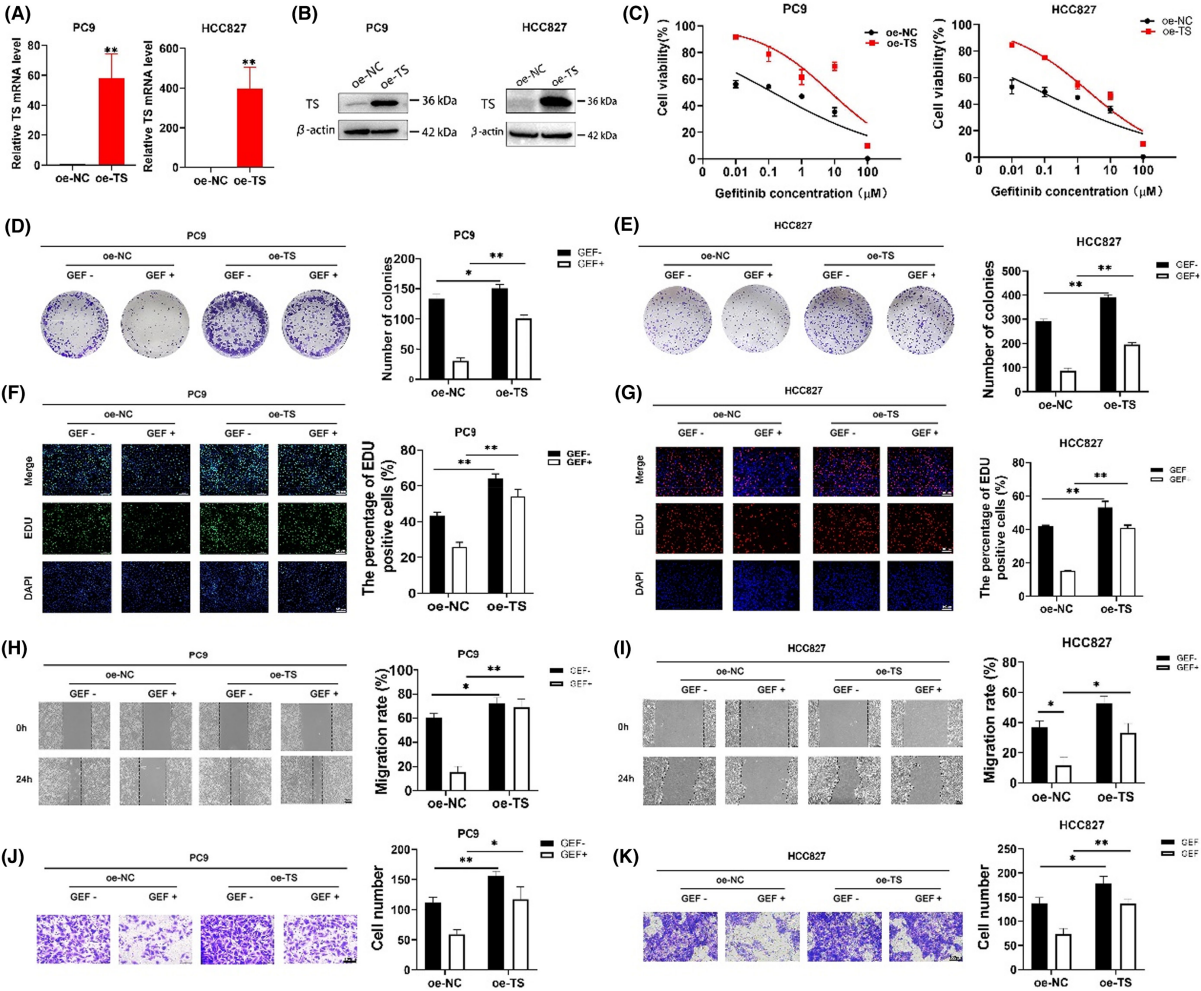
TS overexpression promoted cell proliferation, DNA replication and cell migration in PC9 and HCC827 cells under gefitinib treatment. (A**)**, the TS mRNA level of PC9 and HCC827 cells transfected with overexpressed plasmid of TS (oe‐TS) for 48 h. (B), the TS protein level of PC9 and HCC827 cells transfected with oe‐TS plasmid for 48 h. (C), the IC50 value of gefitinib (GEF) in transfected PC9 and HCC827 cells treated with GEF at gradient concentrations for 48 h. (D, E), colony formation experiments used to estimate the proliferation of PC9 and HCC827 cells transfected with the oe‐TS plasmid with treatment of GEF. (F, G), EdU (green or red) and DAPI (blue) staining assays were conducted to determine the DNA synthesis capacity of the transfected cells under GEF treatment for 48 h. (H, I), wound healing assay used to measure the migration rate of cells transfected with oe‐TS plasmid under GEF treatment for 24 h. J, K, transwell assays used to calculate the number of migrating cells after overexpression of TS under GEF treatment for 24h. All experiments were performed three times. *p < 0.05, **p < 0.01.

To demonstrate whether TS exerts a vital role in gefitinib resistance in NSCLC, PC9 and HCC827 cells were transfected with a TS overexpression plasmid (oe‐TS). qRT‐PCR assays indicated that TS mRNA expression was significantly upregulated in both two cell lines transfected with oe‐TS (Figure 3A). Likewise, western blot experiments showed that TS protein level dramatically increased when transfected with oe‐TS (Figure 3B). CCK8 assays demonstrated that the survival rate of PC9 and HCC827 cells transfected with oe‐TS significantly increased under gefitinib treatment at the same concentration (Figure 3C). The IC50 values of gefitinib in PC9 and HCC827 cells transfected with oe‐TS were over 30‐fold higher than those in cells transfected with oe‐NC. Colony formation ability was considerably stronger after overexpression of TS (Figure 3D,E). Furthermore, ethynyl deoxyuridine (EdU) staining experiments suggested that the upregulation of TS promoted the proliferation of gefitinib‐sensitive cells and enhanced their tolerance to gefitinib (Figure 3F,G).

As tumour metastasis is an essential cause of disease progression and therapy resistance, we hypothesized that TS mediates migration of lung cancer cells during the development of gefitinib resistance. As expected, wound healing assay (Figure 3H,I) and Transwell assay (Figure 3J,K) showed that the migration capacity of PC9 and HCC827 cells with overexpression of TS was increasingly enhanced. Furthermore, the cell migration cannot be hampered under the gefitinib treatment when TS was upregulated in resistant cells.

### 2.5 TS inhibition restores gefitinib sensitivity in gefitinib‐resistant cells

**FIGURE 4:**
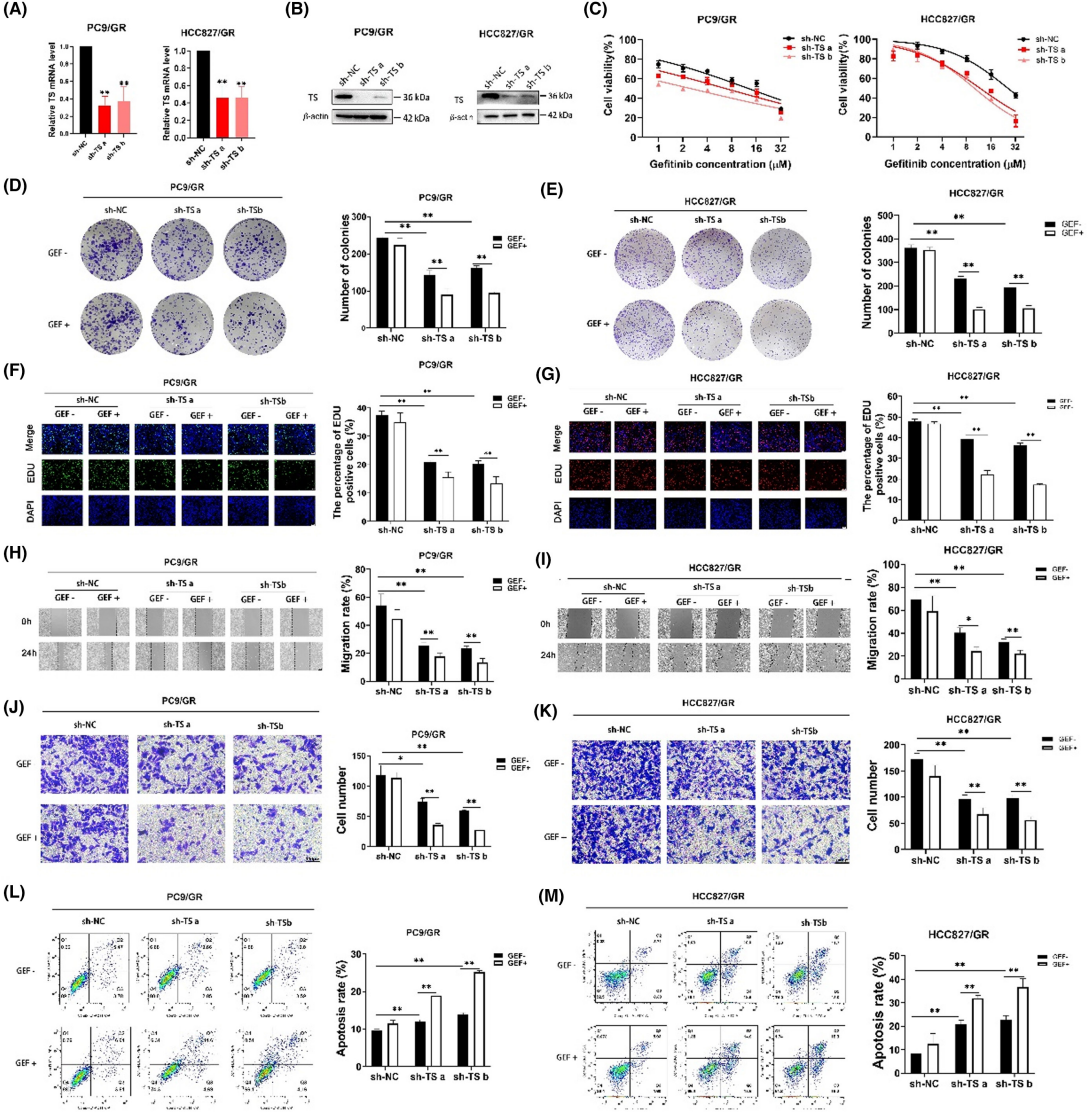
The knockdown of TS suppressed cell survival, hampered DNA replication, inhibited cell migration, and induced cell apoptosis in gefitinib‐resistant cells. (A), the TS mRNA level of resistant cells with knockdown of TS. (B), the protein expression of TS in resistant cells with TS knockdown for 48 h. (C), the IC50 values of resistant cells with TS knockdown were measured by CCK8 assay under the treatment of gefitinib (GEF) for 48 h. (D, E), the colony formation ability of resistant cells after TS knockdown. (F, G), EdU staining assays used to detect DNA replication of resistant cells with TS knockdown in treatment of GEF for 48 h. (H, I), wound healing experiments were performed to evaluate the migrating ability of resistant cells with TS knockdown. (J, K), transwell assays were used to detect the number of cell migration after knockdown of TS. (L, M), flow cytometric analysis of cell apoptosis in resistant cells transfected with TS‐knockout plasmid and in treatment of GEF for 48 h. Three independent experiments were conducted. *p < 0.05, **p < 0.01.

To further verify the above results, we subsequently transfected PC9/GR and HCC827/GR cells with sh‐NC or two different short hairpin RNA targeting TS (sh‐TS a/b). qRT‐PCR assay (Figure 4A) and western blot assay (Figure 4B) were performed to validate the TS knockout efficiency. CCK8 assays showed that knockdown of TS made resistant cells less tolerant to gefitinib (Figure 4C). Figure 4D,E showed the colony number of resistant cells transfected with sh‐TS a/b was dramatically decreased. EdU staining experiments indicated this knockdown inhibited the cell proliferation and the inhibitory effect was further enhanced in the presence of gefitinib (Figure 4,G). Moreover, the function of cell migration was dramatically decreased after the downregulation of TS in resistant cells, and this effect was enhanced under the treatment of gefitinib (Figure 4,I). Conversely, the knockdown of TS‐induced cell apoptosis in gefitinib‐resistant cells and this effect was enhanced with gefitinib treatment (Figure 4J,K). The above results demonstrated that TS may mediate gefitinib resistance, and the downregulation of TS can restore sensitivity in gefitinib‐resistant NSCLC.

### 2.6 Pemetrexed inhibits TS‐mediated thymidylate biosynthesis to restore sensitivity to gefitinib

**FIGURE 5:**
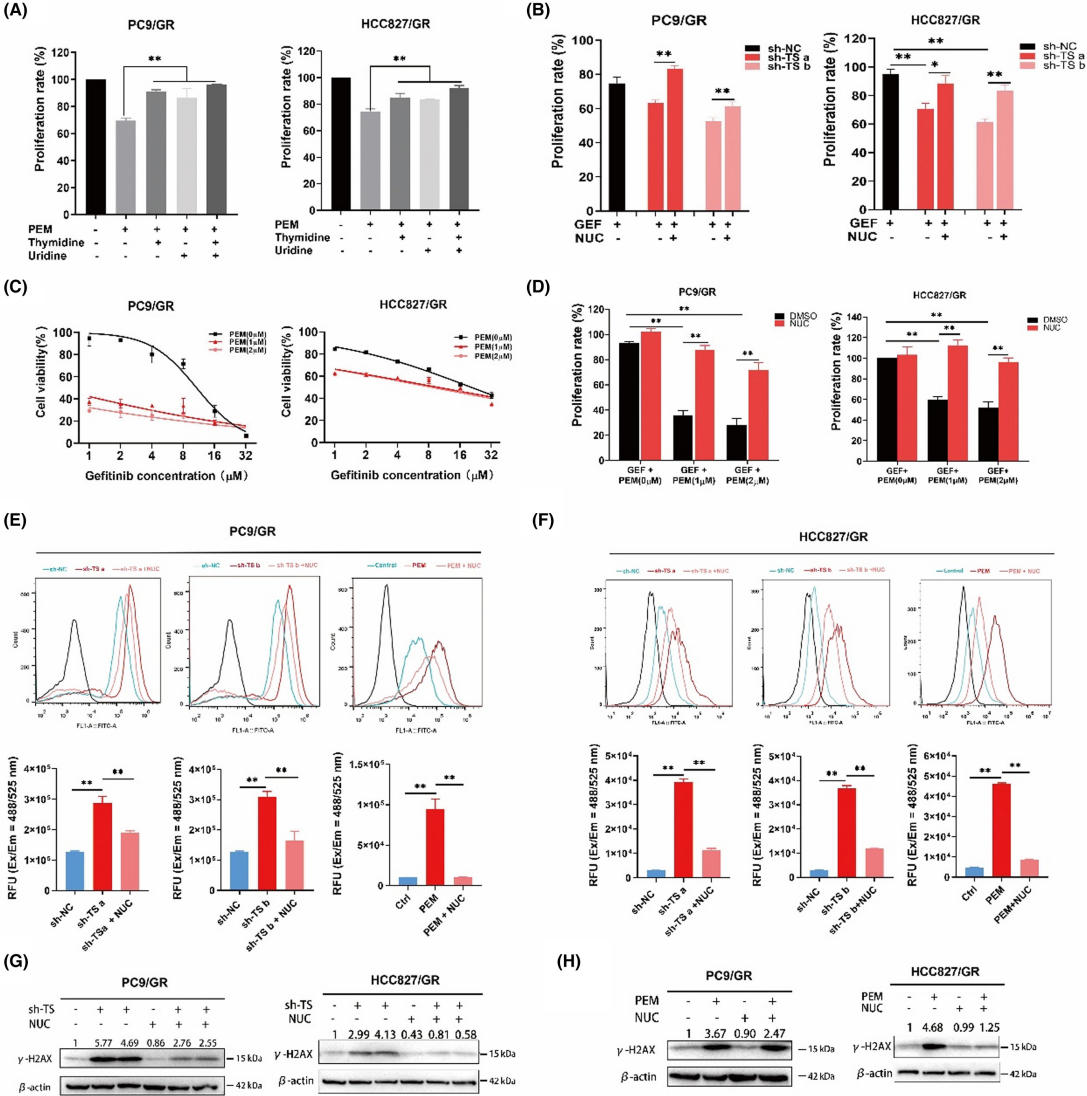
The inhibition of TS and pemetrexed restored the sensitivity of resistant cells to gefitinib through inducing ROS generation and DNA damage. (A), CCK8 assays showed the proliferation rate of resistant cells treated with pemetrexed (PEM) and/or exogenous nucleotide precursors (uridine and thymidine) or their cocktail (NUC). (B), the proliferation ability of resistant cells transfected with TS knockdown was rescued by NUC supplementation. (C), the IC50 values of gefitinib (GEF) in resistant cells treated with PEM for 48 h. (D), the viability of resistant cells cotreated with GEF and PEM for 48 h. (E, F), the knockdown of TS or pemetrexed treatment in resistant cells promoted ROS generation and this effect was rescued by NUC supplementation. The respective histograms constructed based on the quantitative analysis of fluorescence intensity. The fluorescence signal was monitored at Ex/Em = 488/525 nm. RFU, relative fluorescence units. (G), western blot analysis of the expression of γ‐H2AX in resistant cells treated with TS‐knockdown and the supplementation of NUC. (H), the protein level of γ‐H2AX in resistant cells with PEM ± NUC.

Pemetrexed is well known to inhibit TS‐mediated nucleotide biosynthesis to exert antitumor effects and is widely used as a preferable chemotherapeutic agent for numerous malignancies. Our previous studies revealed that a proportion of advanced NSCLC patients who experience disease progression during EGFR‐TKI therapy could benefit from previously administered targeted agents after cisplatin‐pemetrexed chemotherapy.^28^ Thus, we hypothesized that pemetrexed might restore the sensitivity of gefitinib‐resistant NSCLC cells to gefitinib through inhibiting the biological function of TS. We first demonstrated that pemetrexed can inhibit gefitinib‐resistant cell proliferation and this effect could be rescued by the exogenous nucleotide precursors thymidine and uridine. Moreover, combination treatment with thymidine and uridine (NUC) further weakened the cytotoxic effect of pemetrexed in resistant cells (Figure 5A). The common phenomenon was observed after knockdown of TS, and NUC supplementation made resistant cells with TS knockout more tolerant to gefitinib (Figure 5B). CCK8 assays illustrated that the susceptibility of resistant cells to gefitinib was increased after treatment of pemetrexed and this phenomenon displayed a dose–response relationship (Figure 5C). However, this anticancer effect was diminished by exogenous supplementation with NUC (Figure 5D). That is, activated nucleotide metabolism promoted gefitinib resistance in lung cancer, whereas pemetrexed impaired cellular nucleotide metabolism through inhibition of TS, thereby diminishing the cell proliferation and further re‐sensitising the resistant cells to gefitinib.

### 2.7 Pemetrexed inhibits TS‐mediated thymidylate biosynthesis to induce ROS generation and DNA damage

Of note, TS utilizes N 10‐formyl tetrahydrofolate (N 10‐fTHF) as the methyl‐group donor to methylate deoxyuridine‐5′‐monophosphate (dUMP) to deoxythymidine‐5′‐monophosphate (dTMP), and the latter is subsequently converted to deoxythymidine triphosphate (dTTP), which serves as a crucial precursor for DNA replication and damage repair. We next detected intracellular levels of reactive oxygen species (ROS), which represent the cellular ability to clear oxidative damage and repair DNA damage. We found that intracellular ROS levels were significantly elevated in resistant cells treated with either TS knockdown or pemetrexed. However, after exogenous supplementation with nucleotide precursors, the intracellular ROS levels were decreased (Figure 5E,F). As the accumulation of ROS generation is one of major characteristics of DNA damage, we detected the expression of γ‐H2AX, which is a well known indicator in response to DNA damage. The western blot analysis revealed that either the knockdown of TS (Figure 5G) or pemetrexed treatment (Figure 5H) could dramatically enhance the expression of γ‐H2AX. However, the expression of γ‐H2AX could be diminished after supplementation of NUC. These data indicated that impaired thymidylate biosynthesis triggers ROS‐based DNA damage.

### 2.8 Pemetrexed inhibits TS‐mediated thymidylate biosynthesis to promote cellular senescence and the senescence‐associated secretory phenotype (SASP)

**FIGURE 6:**
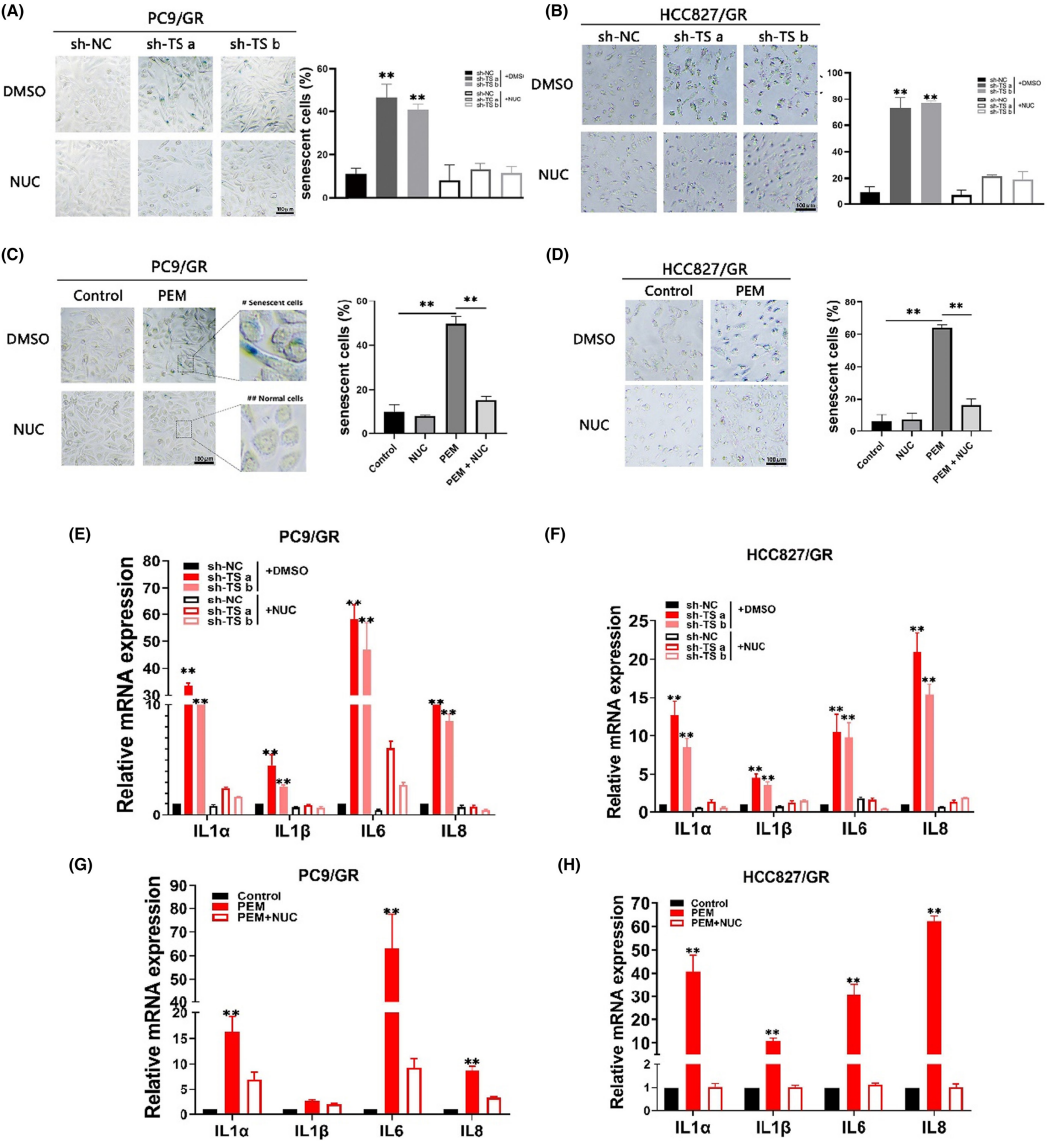
The inhibition of TS and pemetrexed‐induced cellular senescence in resistant cells. (A, B), SA‐β‐Gal staining assays in resistant cells after treatment with TS‐knockdown and the supplementation of NUC. The blue cells represented senescent cells. (C, D), SA‐β‐Gal staining assays in resistant cells with PEM±NUC treatment. E and F, the mRNA levels of SASP‐associated genes in resistant cells with TS‐knockdown and the supplementation of NUC. (E, F), the mRNA levels of SASP‐associated genes in resistant cells with PEM ± treatment. Each experiment was conducted three times independently. **p < 0.01.

The effects of increased ROS level and DNA damage observed in the above experiments triggered us to evaluate the induction of cellular senescence. We first examined senescence‐associated β‐galactosidase (SA‐β‐Gal) activity in gefitinib‐resistant cells after TS knockdown or in treatment with pemetrexed. SA‐β‐Gal staining showed that the proportion of SA‐β‐Gal‐positive cells was higher after TS knockdown (Figure 6A,B) or pemetrexed treatment (Figure 6C,D). The picture marked with # in Figure 6C visually displays the morphological changes related to the senescence response in resistant cells, such as enlarged cell size, more irregular protrusions and intensive cytoplasmic vacuolisation. In addition, plenty of inflammatory cytokines considered as the senescence‐associated secretory phenotype (SASP), such as IL‐1α, IL‐1β, IL‐6 and IL‐8, showed a similar upregulation pattern in gefitinib‐resistant cells (Figure 6E–H). Conversely, the effect of cellular senescence triggered by pemetrexed or TS knockdown could be eliminated by exogenous supplementation with NUC. All the above results suggested that the damaged nucleotide biosynthesis induced by pemetrexed or knockdown of TS leads to a lack of nucleotide materials and higher intercellular replication stress, thus, cells fail to defend against ROS‐based DNA damage and fate to cellular senescence.

### 2.9 Gefitinib combined with pemetrexed attenuates resistant cell progression

**FIGURE 7:**
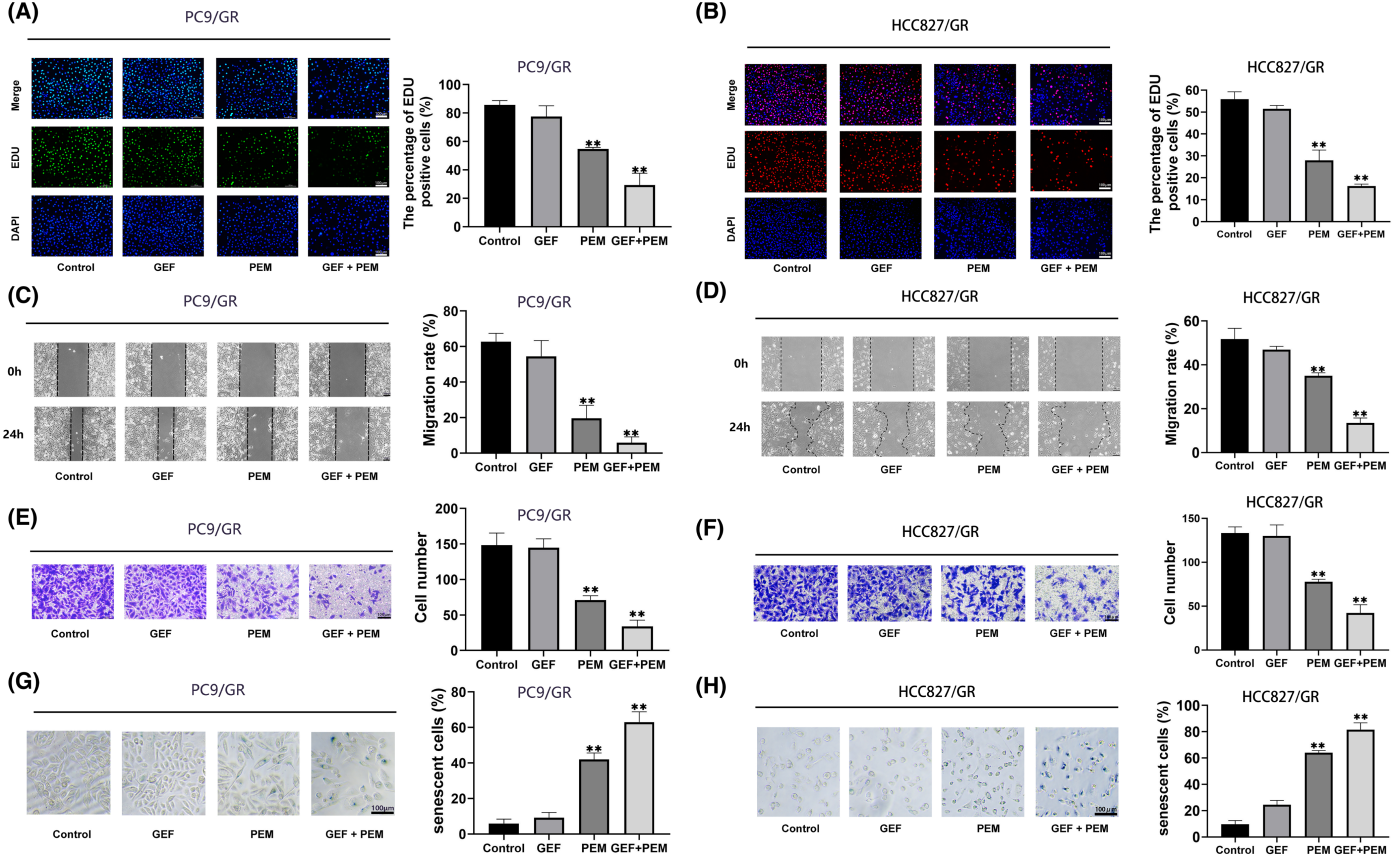
Gefitinib (GEF) and pemetrexed (PEM) combination treatment synergistically induced cellular senescence and hampered cell survival in gefitinib‐resistant cells. (A, B), EdU staining assays illustrated DNA proliferation in resistant cells treated with GEF and/or PEM for 48 h. The number of proliferating cells was counted with ImageJ and is displayed as the percentage of proliferating cells. (C, D**)**, the migration of resistant cells treated with GEF and/or PEM for 48 h was evaluated with wound healing assays. The migration rate = healing area of scratch/initial area of scratch × 100%. (E, F), transwell assays of resistant cells under the above treatments. (G, H), SA‐β‐Gal staining assays in resistant cells treated with GEF and/or PEM. Histogram analysis showing the results from three independent experiments. **, *p* < 0.01.

Based on the above results, we attempted to explore the synergistic anticancer activity of pemetrexed and gefitinib (GP) in gefitinib‐resistant cells. EdU staining assays illustrated that GP treatment significantly hampered DNA proliferation to a greater extent than pemetrexed or gefitinib alone (Figure 7A,B). Functionally, we subsequently verified the migrating potential of resistant cells treated with GP. Wound healing assay (Figure 7C,D) and Transwell assays (Figure 7E,F) suggested that the cells displayed a decreased migration rate after GP treatment compared with single‐drug treatment. Furthermore, more senescent cells were induced in resistant cells treated with GP (Figure 7G,H). These data revealed that pemetrexed can inhibit TS‐mediated nucleotide biosynthesis, thereby inducing cellular senescence, thereafter damaging the function of cell proliferation and survival. Pemetrexed combined with gefitinib still exhibited synergistic antitumor effect in gefitinib‐resistant NSCLC.

## 3 DISCUSSION

As an EGFR‐targeted small molecule agent, gefitinib has brought unprecedented therapeutic benefits in NSCLC patients with driving mutations in the EGFR gene, including exon 19 deletion and L858R mutation. Unfortunately, the efficacy of gefitinib is inevitably diminished due to the emergence of acquired resistance during the course of treatment. The molecular mechanism determining EGFR‐TKI resistance in lung cancer remains incompletely understood. In our study, we first illustrated the role of TS‐mediated thymidylate nucleotide biosynthesis in the development of gefitinib resistance. We demonstrated that NSCLC patients with higher expression of TS gene have shorter PFS during EGFR‐TKI treatment. Furthermore, we found TS is upregulated in NSCLC patients resistant to gefitinib and in PC9/GR cells which are tolerant of gefitinib. Knockdown of TS‐induced apoptosis and diminished survival of gefitinib‐resistant NSCLC.

Nucleotides are the dominant genetic biosynthesis building blocks and consist of pyrimidines (thymine, uracil and cytosine) and purines (adenine and guanine). These substances are vital for DNA and RNA biosynthesis, cell fate signalling, enzyme function regulation and cell metabolism. The synthesis and availability of large amounts of nucleotides and energy are necessary to support the growth and survival of malignant cells, and accelerated de novo nucleotide metabolism provides abundant nucleotide materials for cell proliferation, which suggests that nucleotide metabolism is a promising target for anticancer treatment.^33^ As a key enzyme in the thymidylate metabolism, TS maintains the dTMP (thymidine‐5‐prime monophosphate) pool critical for DNA replication and repair. In this study, we concentrated on the function of TS on the cell fate changes. We reported that knockdown of TS triggered ROS generation, DNA damage and cellular senescence, which might explain the inhibition of TS‐impaired gefitinib‐resistant cell proliferation, facilitated cell apoptosis, as well as hampered cell migration. Cellular senescence is widely recognized as a potent tumour‐suppressing mechanism. Pemetrexed is a multitargeted antifolate that inhibits TS in addition to multiple other folate‐dependent enzymes. We demonstrated the inhibition of TS is the predominant mechanism of pemetrexed‐mediated anti‐proliferative effects, as this outcome can be markedly attenuated with supplementary of exogenous nucleotide precursors. Interestingly, pemetrexed showed the potential of promoting ROS‐based DNA damage and cellular senescence in gefitinib‐resistant NSCLC. The lack of thymidine nucleotide production after TS knockdown or pemetrexed treatment diminished gefitinib‐resistant cell survival. In addition, the therapeutic strategy of gefitinib in combination with pemetrexed exhibiting an excellent anticancer effect in gefitinib‐resistant cells, indicating that pemetrexed could reverse gefitinib resistance through damaging TS‐mediated thymidine nucleotide metabolism. In conclusion, our present study illustrated that NSCLC patients with high TS expression are more inclined to develop resistance during EGFR‐TKI‐targeted therapy. Concomitant pemetrexed administration can retard the evolution of resistance and restore the sensitivity of cancer cells to EGFR‐TKI. This study provides a compelling rationale for selecting an advantageous population for targeted agents combined with chemotherapy, which has significant clinical implications and far‐reaching practical value.

## 4 MATERIALS AND METHODS

### 4.1 Patient characteristics and tissue samples for survival analysis

A total of 140 patients were enrolled in this study. Information about EGFR mutations, TS polymorphism variants and the clinicopathological characteristics of NSCLC patients is summarized in Table 1. For pathological sample collection, a total of 24 LUAD patients harbouring either EGFR exon 19DEL or L858R were enrolled in the study. The project was approved by the Research Ethics Committee of Nanjing Medical University (Nanjing, Jiangsu, China), and written informed consent was obtained from all patients.

### 4.2 Cell culture

The human NSCLC cell lines PC9, HCC827 (EGFR exon 19 deletion) and gefitinib‐resistant cell lines PC9/GR, HCC827/GR (no T790M mutation) were purchased from the Institute of Biochemistry and Cell Biology at the Chinese Academy of Sciences (Shanghai, China). The cells were cultured in DMEM (Gibco‐BRL) containing 10% foetal bovine serum (FBS; Gibco) and 1% penicillin–streptomycin (Gibco) at 37°C in incubators with 5% CO_2_.

### 4.3 Quantitative real‐time PCR (qRT‐PCR)

Total RNA was isolated from tissues or cells using TRIzol reagent (Thermo Fisher Scientific). Then, 1.0 μg of isolated RNA was reverse‐transcribed to cDNA with PrimeScript™ RT reagent (Takara, Japan) according to the manufacturer's instructions. qRT‐PCR analysis was performed with SYBR Green (Takara, Japan). In accordance with the manufacturer's instructions, qRT‐PCR and data collection were carried out with a StepOnePlus RT–PCR system (Applied Biosystems). GAPDH was used as the normalisation control. The sequences of all primers used in this study are listed in Table S1.

### 4.4 Western blot analysis and antibodies

Cell proteins were lysed with RIPA buffer (Sigma) and subsequently subjected to ultrasonic lysis. Total protein was separated in a 10% SDS–PAGE gel and transferred to a PVDF membrane (Millipore, USA). After incubation with antibodies and washing, signals were detected using a chemiluminescence system (Bio‐Rad). The primary antibodies used in this study were anti‐TS (cat no. 15047‐1‐AP, Proteintech, China) and anti‐β‐actin (cat no. 66009‐1‐IG, Proteintech) and anti‐γ‐H2AX (sc‐517,336; Santa Cruz Biotechnology).

### 4.5 Transfection of cell lines

The empty vector plasmid (oe‐NC), TS‐overexpressing plasmid (oe‐TS) and two different short hairpin RNAs against TS (sh‐TS a/b) were synthesized by Genscript Corporation (Nanjing, China). The ORF Sequence Information for TS and the targeted sequences for sh‐TS a/b are listed in Table S2. Lipofectamine 3000 transfection reagent (Thermo Fisher Scientific) was used for cell transfection according to the manufacturer's instructions. After transfection for 48 h, the cells were harvested and processed for further experiments.

### 4.6 CCK8 assay

The survival rate of cells treated with gefitinib was estimated using CCK8 assays (Selleck, Shanghai, China). Cells transfected with specific plasmid were seeded into 96‐well plates at 3000 cells per well and exposed to different concentrations of gefitinib (MedChemExpress, China) for 48 h. After treatments, 10 μL of CCK8 was added to each well and incubated with the cells at 37°C for 1 hour. The absorbance was measured using an enzyme microplate reader at 450 nm. Each treatment was applied in triplicate, and three independent experiments were performed.

### 4.7 Colony formation assay

Cells were collected after the above‐mentioned treatments and reseeded into 6‐well plates at 1000 cells/well. Then, the cells were treated with the corresponding drug or PBS for 24 h. The medium was changed every 3 days. After incubation for 14 days, the cells were fixed with paraformaldehyde (4%) for 30 min and subsequently stained with crystal violet (0.5%) for 30 min. After a brief wash with PBS, the plates were allowed to dry at room temperature, and colonies were photographed. Each experiment was performed in triplicate.

### 4.8 Ethynyl deoxyuridine (EdU) staining analysis

A BeyoClick™ EdU Cell Proliferation Kit with Alexa Fluor 488/555 (Beyotime, Shanghai, China) was used to assess cell proliferation. The cells were seeded in 96‐well plates at a density of 5000 cells/well. After transfection or different drug treatments, 10 μM EdU labelling medium was added to the 96‐well plates and incubated with the cells for 2 h at 37°C under 5% CO_2_. After that, the cells were treated with 4% paraformaldehyde and 0.3% Triton X‐100 and subsequently stained with anti‐EdU working solution. Hoechst 33342 was used to label the cell nuclei. The percentage of EdU‐positive cells was estimated after fluorescence microscopy analysis.

### 4.9 Wound healing assay

The indicated cells were cultured on 6‐well plates at 5 × 10^5^ cells per well and grown to total confluence for 24 h. A pipette tip (200 μL) was used to scratch three vertical lines in each well. At 0 and 24 h, images of the scratches were taken. The healing area of the scratches was calculated using ImageJ software. The experiment was performed in triplicate, and the mean value was calculated. Five fields (200× magnification) of view were randomly selected for imaging and counting.

### 4.10 Transwell assay

In total, 5 × 10^4^ cells in serum‐free medium were placed into the upper chamber of a transwell assay insert (8‐μm pore size, Millipore). Medium containing 10% FBS and 1 μmol/L gefitinib and/or pemetrexed was added to the lower chamber. After culturing for 24 h, cells that invaded through the membrane were fixed with 4% paraformaldehyde for 15 minutes and stained with crystal violet for 10 minutes, while cells that had not migrated or invaded the membrane were removed with cotton swabs. Cells were imaged using an IX71 inverted microscope (Olympus, Tokyo, Japan) at 200× magnification.

### 4.11 Flow cytometric analysis of cell apoptosis

Cells transfected with sh‐TS or sh‐NC were treated with gefitinib and cultured for 48 h. For cell apoptosis analysis, the cells were harvested by trypsin without EDTA and double stained with Annexin V‐Alexa Fluor 647 and propidium iodide using the Annexin V‐Alexa Fluor 647/PI apoptosis detection kit (YEASEN). Cell apoptosis ratio was analysed by a flow cytometer (FACScan, BD Biosciences). Cells were divided into viable cells, dead cells, early apoptotic cells and apoptotic cells. The relative apoptotic ratios of cells with gefitinib treatment were compared with cells without gefitinib treatment.

### 4.12 ROS detection

Cells were treated with 10 μM DCFDA (2′,7′‐dichlorodihydrofluorescein) for 30 min in the dark. After two washes with PBS and trypsinisation for collection, the cells were immediately analysed via flow cytometry. Intracellular DCFDA signals were measured with a flow cytometer (FACScan, BD Biosciences). ROS levels are shown as relative fluorescence units (RFU) at Ex/Em = 488/525 nm.

### 4.13 Senescence‐associated β‐Gal assay

Cells transfected with sh‐TS or treated with pemetrexed for 48 h were plated in 12‐well plates. The chromogenic β‐gal substrate X‐gal (C0602, Beyotime Biotechnology) was used to stain the fixed cells for 8 h at 37°C. The cells were washed three times with PBS, and microscopy images were taken by bright field microscopy at 100× magnification.

### 4.14 Statistical analysis

The data were analysed using GraphPad Prism 8 software (GraphPad Software, La Jolla, CA, USA). PFS was estimated using Kaplan–Meier analysis, and differences were determined with a log‐rank test. Differences between groups were compared using a *t* test, and a *p* value <0.05 was considered statistically significant.

## AUTHOR CONTRIBUTIONS

**Yun Chen:** Conceptualization (lead); methodology (lead); resources (equal); visualization (lead); writing – original draft (lead); writing – review and editing (lead). **Chen Zhang:** Data curation (equal); software (equal); validation (equal). **Shidai Jin:** Formal analysis (equal); resources (equal). **Jun Li:** Formal analysis (equal); resources (equal). **Jiali Dai:** Validation (supporting). **Zhihong Zhang:** Investigation (equal); project administration (equal). **Renhua Guo:** Funding acquisition (equal); investigation (equal); project administration (equal); supervision (equal).

## CONFLICT OF INTEREST STATEMENT

The authors report no competing interests related to this study.

## Supporting information


Tables S1–S2

## Data Availability

The data that support the findings of this study are available from the corresponding author upon reasonable request.
