# Supplementary material for: Pemetrexed induces ROS generation and cellular senescence by attenuating TS‐mediated thymidylate metabolism to reverse gefitinib resistance in NSCLC
Source: J Cell Mol Med. 2023 Jun 6;27(14):2032–44. doi: 10.1111/jcmm.17799 (PMC10339075; doi:10.1111/jcmm.17799)
Supplement: Supplementary file 1 — Tables S1–S2 [file JCMM-27-2032-s001.pdf]

Table S1. Primer sequences used in this study.

Name	Primer
GAPDH F	TGGTCACCAGGGCTGCTT
GAPDH R	AGCTTCCCGTTCTCAGCCTT
TS F	GGGACTTGGGCCCAGTTTAT
TS R	CTTCTGTCGTCAGGGTTGGT
IL-1α F	AGTAGCAACCAACGGGAAGG
IL-1α R	AAGGTGCTGACCTAGGCTTG
IL-1β F	GCCATGGACAAGCTGAGGAA
IL-1β R	TCGTTATCCCATGTGTCGAAGA
IL6 F	ACTCACCTCTTCAGAACGAATTG
IL6 R	CCATCTTTGGAAGGTTCAGGTTG
IL8 F	CTGAGAGTGATTGAGAGTGGAC
IL8 R	ACCCTCTGCACCCAGTTTTC

Table S2. ORF sequences for TS over expression and targeted sequences for TS knockdown.

Clone Name	Target Sequence
ORF Sequence Information for TS	ATGCCTGTGGCCGGCTCGGAGCTGCCGCGCCGGCCCTTGCCCCCCGCCGCACAG<br>GAGCGGGACGCCGAGCCGCGTCCGCCGCACGGGGAGCTGCAGTACCTGGGGCAG<br>ATCCAACACATCCTCCGCTGCGGCGTCAGGAAGGACGACCGCACGGGCACCGGC<br>ACCCTGTCGGTATTCGGCATGCAGGCGCGCTACAGCCTGAGAGATGAATTCCCT<br>CTGCTGACAACCAAACGTGTGTTCTGGAAGGGTGTTTTGGAGGAGTTGCTGTGG<br>TTTATCAAGGGATCCACAAATGCTAAAGAGCTGTCTTCCAAGGGAGTGAAAATC<br>TGGGATGCCAATGGATCCCGAGACTTTTTGGACAGCCTGGGATTCTCCACCAGA<br>GAAGAAGGGGACTTGGGCCCAGTTTATGGCTTCCAGTGGAGGCATTTTGGGGCA<br>GAATACAGAGATATGGAATCAGATTATTCAGGACAGGGAGTTGACCAACTGCAA<br>AGAGTGATTGACACCATCAAAACCAACCCTGACGACAGAAGAATCATCATGTGC<br>GCTTGGAATCCAAGAGATCTTCCTCTGATGGCGCTGCCTCCATGCCATGCCCTC<br>TGCCAGTTCTATGTGGTGAACAGTGAGCTGTCCTGCCAGCTGTACCAGAGATCG<br>GGAGACATGGGCCTCGGTGTGCCTTTCAACATCGCCAGCTACGCCCTGCTCACG<br>TACATGATTGCGCACATCACGGGCCTGAAGCCAGGTGACTTTATACACACTTTG<br>GGAGATGCACATATTTACCTGAATCACATCGAGCCACTGAAAATTCAGCTTCAG<br>CGAGAACCCAGACCTTTCCCAAAGCTCAGGATTCTTCGAAAAGTTGAGAAAATT<br>GATGACTTCAAAGCTGAAGACTTTCAGATTGAAGGGTACAATCCGCATCCAACT<br>ATTAAAATGGAAATGGCTGTTTAG
sh-TS a	GGGATTCTCCACCAGAGAAGA
sh-TS b	GACAACCAAACGTGTGTTCTG
